# BRAF^V600E^ hot spot mutation in thyroid carcinomas: first Moroccan experience from a single-institution retrospective study

**DOI:** 10.4314/ahs.v20i4.40

**Published:** 2020-12

**Authors:** Meryem Kaabouch, Hafsa Chahdi, Naima Azouzi, Mohammed Oukabli, Issam Rharrassi, Adil Boudhas, Hassan Jaddi, Mouna Ababou, Nadia Dakka, Amélie Boichard, Youssef Bakri, Corinne Dupuy, Abderrahmane Al Bouzidi, Rabii Ameziane El Hassani

**Affiliations:** 1 Laboratory of Biology of Human Pathologies “BioPatH”, Center for Genomics of Human Pathologies “GenoPatH”. Faculty of Science in Rabat. Mohammed V University in Rabat, Morocco; 2 Anatomic Pathology and Histology Service, Military Hospital Mohammed V of Rabat, Morocco. Equipe de Recherche en PathologieTumorale. Faculty of Medicine and Pharmacy of Rabat, University Mohammed V of Rabat; 3 Faculty of Sciences in Rabat. Centre National de l'Energie, des Sciences et Techniques Nucléaires, Rabat, Morocco; 4 UMR 8200 CNRS, Institut Gustave Roussy, Villejuif, France; 5 Center for Personalized Cancer Therapy. UCSD Moores Cancer Center. 3855 Health Sciences Drive. La Jolla, CA 92093

**Keywords:** Biomarker, BRAF^V600E^, Thyroid cancer, Morocco

## Abstract

**Background:**

The incidence of thyroid cancer is increasing worldwide at an alarming rate. BRAF^V600E^ mutation is described to be associated with a worse prognostic of thyroid carcinomas, as well as extrathyroidal invasion and increased mortality.

**Objective:**

To our knowledge, there are no reported studies neither from Morocco nor from other Maghreb countries regarding the prevalence of BRAF^V600E^ mutation in thyroid carcinomas. Here we aim to evaluate the frequency of BRAF^V600E^ oncogene in Moroccan thyroid carcinomas.

**Methods:**

In this Single-Institution retrospective study realized in the Anatomic Pathology and Histology Service in the Military Hospital of Instruction Mohammed V ‘HMIMV’ in Rabat, we report, using direct genomic sequencing, the assessment of BRAF^V600E^ in 37 thyroid tumors.

**Results:**

We detected BRAF^V600E^ mutation exclusively in Papillary Thyroid Carcinomas ‘PTC’ with a prevalence of 28% (8 PTC out 29 PTC). Like international trends, Papillary Thyroid Carcinomas ‘PTC’ is more frequent than Follicular Thyroid Carcinomas ‘FTC’ and Anaplastic Thyroid Carcinomas ‘ATC’ (29 PTC, 7 FTC and 1 ATC).

**Conclusion:**

Our finding gives to the international community the first estimated incidence of this oncogene in Morocco showing that this prevalence falls within the range of international trends (30% to 90%) reported in distinct worldwide geographic regions.

## Introduction

Thyroid cancer is the commonest malignant endocrine tumour. Follicular thyroid cells (thyrocytes) represent the major part of thyroid cells, and tumors derived from these cells are classified depending on their histological and genetic characterization, in Follicular Thyroid Carcinomas ‘FTC’, Papillary Thyroid Carcinomas ‘PTC’ and undifferentiated Anaplasic Thyroid Carcinomas ‘ATC’^1^. PTC is the major form of thyroid carcinomas accounting more than 80%; and BRAF^V600E^ hot spot mutation is the most frequent genetic alteration of the MAPK pathway in PTC 1; 2; 3; 4; 5; 6; 7.

RAF proteins (A, B and C), serine threonine kinases, are key regulators of the mitogen-activated protein kinase (MAPK) pathway. BRAF mutations have been identified in various cancers with high frequency in melanomas (50–60%) and thyroid cancers (30–90%); and BRAF mutations are mostly V600E substitution 4; 8; 9; 10; 11; 12; 13; 14; 15.

A thymidine-to-adenosine transversion at exon 15 nucleotide 1799 (T1799A) of the BRAF gene, causing a valine-to-glutamic acid change in codon 600 of the BRAF protein, the most common mutation of BRAF, leads to a constitutive activation of MEK/ERK pathway independently of RAS activation. BRAF^V600E^ mutation is associated with a high level of ERK signature because the mutated form of BRAF^V600E^ does not respond to the negative feedback of ERK 5; 16.

Several studies have investigated the clinical significance of BRAF^V600E^ mutation in thyroid carcinomas and its role as diagnostic and prognostic marker remains unclear^1^; ^17^; ^18^.

Molecular assessment of BRAF^V600E^ mutation, frequently found in classical forms of PTC and rare in follicular variant forms of PTC (FVPTC), might in some cases distinguish between PTC and FVPTC overcoming the ambiguity of the histological and cytological diagnosis^1^; ^19^. Also, the presence of BRAF^V600E^ mutation might discriminate between benign and malignant thyroid nodules during a fine-needle biopsy (FNB) cytological examination 1; 20. In fact, molecular detection of BRAF^V600E^, as an early event in thyroid tumorigenesis, could be particularly helpful for clinicians to improve diagnosis of thyroid nodules sampled by FNB and classified by cytology as indeterminate for malignancy or AUS/FLUS “atypia of undetermined significance/follicular lesion of undetermined significance. Although the single mutational testing for BRAF^V600E^ has high specificity for thyroid malignancy, the 2015 ATA guidelines do not recommended the systematic use of the single molecular status of BRAF; and a mutational panels (RAS, BRAF, RET/PTC, ..) might be most helpful for appropriate and individual management of thyroid carcinomas (Haugen BR et al., 2015 ATA guidelines). Concerning the clinical use of BRAF^V600E^ as prognostic marker, conflicting conclusions are deduced from the literature 1; 18. Owing to the ability of BRAF^V600E^ oncogene to predispose thyroid tumors to dedifferentiation, and knowing that thyroid de differentiation leads to the resistance to radioiodine therapy (I131) of thyroid tumors, BRAF^V600E^ is thereby considered as one of the most powerful prognostic marker for thyroid carcinomas. PTC tumors harboring BRAF^V600E^ show often a loss/decrease expression of the Natrium Iodide Symporter (NIS) which plays a central role in the treatment of thyroid cancer by radioiodine therapy (I131); and PTC-BRAF^V600E^ tumors are reported to be refractory to radioiodine therapy 5; 6; 21; 22. In the same way, a retrospective study of 1849 patients followed and treated for PTC concluded that BRAF^V600E^ mutation was significantly associated with increased cancer-related mortality among patients with PTC 4. In addition, several studies have shown that BRAF^V600E^ oncogene is associated with extrathyroidal invasion, dedifferentiation, loss of radioiodine avidity, and resistance to radioiodine therapy^5^; ^6^; ^7^; ^21^; ^22^; ^23^. However, other groups did not find association between BRAF^V600E^ and worse prognostic and mortality in thyroid cancer 12; 17; 24; 25 . Using a single stranded conformation polymorphism followed by direct sequencing, any association between BRAF^V600E^ mutation and tumor aggressiveness has been observed 17; and using pyrosequencing, Barbaro D et al., deduced that BRAF^V600E^ mutation is not associated with a worse prognosis.

According to the literature, this apparent discrepancy, concerning the clinical significance of BRAF^V600E^ mutation, could be explained by numerous parameters including particularly 1) the detection method used for BRAF^V600E^ testing and 2) the activation threshold of ERK signalling pathway in thyroid tumors harboring BRAF^V600E^.

Unlike direct sequencing of BRAF^V600E^, the quantitative sequencing approach ‘pyrosequencing’ that allow the determination of the ratio of BRAF^V600E^ /BRAFwt is usually advocated for thyroid tumors management 26; 25. Higher prevalence of BRAF^V600E^ mutation has been observed with pyrosequencing compared to direct sequencing^27^; ^28^; and the authors recommended the quantitative preoperative analysis of BRAF^V600E^ by pyrosequencing, which could refine the PTC risk stratification. Marotta V et al., discussed the limitation of qualitative BRAF^V600E^ determination and highlighted the additional value of the quantitative detection of BRAF^V600E^ mutation that could correlate the presence of BRAF^V600E^ to the threshold of mutated alleles associated with a poor prognostic 18.

The incidence of thyroid cancer, especially PTC, is increasing worldwide at an alarming rate; and by 2019, papillary thyroid cancer will double in incidence and become the third most common cancer in women in the United States of America 29; 30. Thereby, thyroid cancer is increasingly a major public health issue, particularly for women. According to the Casablanca registry (2005–2007; Morocco) and Rabat Cancer Registry (2006–2008; Morocco), thyroid malignancies are classified respectively in the third (6.7 per 100 000 persons) and in the fifth range (3.9 per 100 000 persons) 31; 32; 33. This alarming incidence illustrate that thyroid cancer is one of the most frequent female cancers in Morocco. The population-based cancer registry of Casablanca and Rabat are the two principal cancer registries in Morocco including different types of useful data (global incidence and mortality of each cancer, Age-standardized Incidence and Mortality, survival analysis…). There are no reported studies neither from Morocco nor from other Maghreb countries regarding the prevalence of BRAF^V600E^ mutation in thyroid carcinomas. In this retrospective Single-Institution study, we analyzed the prevalence of BRAF^V600E^ hot spot mutation in 37 human thyroid tumors blocks archived in the Anatomic Pathology and Histology Service in the Military Hospital of Instruction Mohammed V ‘HMIMV’ in Rabat, Morocco. We found that, like international trends, this mutation occurs exclusively in PTC tumors and that its prevalence (28%) falls within the range of international trends.

## Patients and methods

### Samples

Formalin-fixed paraffin-embedded (FFPE) thyroid tumor blocks: This retrospective study is carried out according to the approval local ethical committee of the Faculty of Medicine and Pharmacy in Rabat. We have included all available thyroid tumors blocks showing at the minimum 50% of estimated tumor cell percentage after HE staining ‘Hematoxylin and Eosin’. All thyroid cancer cases are arising from follicular origin and operated at the Military Hospital of Instruction Mohammed V ‘HMIMV’ in Rabat between January 1999 and December 2012. After collection of all available clinical informations from registries, and after estimation of the tumor cell percentage by the experimented Pathologists from Anatomic Pathology and Histology Service in the HMIMV of Rabat, only 37 thyroid tumors responding to our inclusion criteria (availability and tumor cell percentage) are chosen for genomic DNA extraction.

BCPAP Cell line: derived from human PTC (heterozygous for BRAF^V600E^) is cultured as described in Dulbecco's modified Eagle's medium (DMEM) (4.5 g/l glucose) (Life Technologies) supplemented with 10% (vol/vol) FCS (Life Technologies) and penicillin/streptomycin (100 mg/ml; Life Technologies) as previously described 22.

### Histological classification

Thyroid tumors are histologically classified by experimented pathologists according to the World Health Organization (WHO) classification after Hematoxylin and Eosin Staining (Lloyd R. V et al., 4^th^ Edition. IARC: Lyon 2017).

### Genomic DNA extraction

After collection of 4 sections of FFPE tissue in Eppendorf tubes (5 µm/section), samples were deparaffinized and digested with proteinase K at 56°C overnight. Genomic DNA was extracted using Bioline kit according to manufacture's protocol; and the quality of extracted DNA was checked using the Qubit fluorometer. It is important to note that the experimented pathologists make one staining HE before and after cutting sections for genomic DNA to be sure that extracted genomic DNA is from the part of tumor blocks containing at the minimum 50% of estimated tumor cell percentage.

### BRAF^V600E^ detection

For BRAF^V600E^ detection, we have used the standard sequencing of BRAF^V600E^ (Exon 15) hot spot mutation routinely used in Gustave Roussy Institute in France: Targeted sequencing for BRAF^V600E^ mutations using Sanger direct sequencing.

Forward tagged primer for BRAF Ex15 amplification is ACCGTTAGTTAGCGATT-TTCATAATGCTTGCTCTGATAGGAAA: Forward primer tag is ACCGTTAGTTAGCGATT and Forward primer that amplified targeted sequence of BRAF Exon 15 is TTCATAATGCTTGCTCTGATAGGAAA.

Reverse tagged primer for BRAF Ex15 amplification is CGGATAGCAAGCTCG-TAGTAACTCAGCAGCATCTCAGGG: Reverse primer tag is CGGATAGCAAGCTCG; and Reverse primer that amplified targeted sequence of BRAF Exon 15 is TAGTAACTCAGCAGCATCTCAGGG.

PCRs were conducted using the HotStart Taq polymerase from Qiagen following cycling conditions: 97°C, 15 min, (97°C, 45 s, 55°C, 30 s, 72°C, 1 min) × 40 cycles, 72°C, 10 min.

The PCR products are purified using the Exo-SAP prior sequencing with the Big Dye Terminator sequencing kit (Applied Biosystems, Foster City, CA). It is important to note that PCR products were bidirectionally sequenced using primers complimentary to the Forward and Reverse tags. The products were analyzed on an automated 3730 DNA Analyzer (Applied Biosystems). Sequence reading and alignment were performed with the SeqScape1 software (Applied Biosystems). The positive samples for BRAF^V600E^ are confirmed with another experimentation (PCR and sequencing).

DNA extracted from human thyroid BCPAP cell line (BRAF^V600E^ /^V600E^ ) is used as positive control for BRAF^V600E^ detection.

### Exploration of data from The Cancer Atlas Genome (TCGA)

Based on the conventional classification, several subtypes of PTCs are grouped in the same group. Recently, a comprehensive multiplatform analysis of homogenous cohort of 496 PTCs developed from The Cancer Atlas Genome (TCGA) has been performed, and allows reclassification of papillary thyroid cancers into molecular subtypes (5). This cohort offers a better understanding and clustering of PTC disease based on Thyroid Differentiation Score (TDS), BRAF-RAS Score (BRS), downstream signalling pathway activated by each pathogenic mutation, and risk assessment. We explore this large cohort of PTC samples from TCGA concerning the prevalence of BRAF^V600E^ mutation and the risk of tumor recurrence already performed from TCGA.

## Results and discussion

Trends by sex and age and Histological types: After clinical registries analysis, checking the availability of tumor blocks and estimation of tumor cell percentage in each available FFPE block, only 37 cases were included in this study. The results summarized in Table 1 show that thyroid carcinomas is more frequent in female confirming what was reported in literature (around three times more thyroid cancers in women than in men) 33; 34; 35. Furthermore, thyroid cancer is diagnosed in older age in men compared to women (50.9±6.1 Vs 42.6±2.1) (Table 1).

**Table 1:** Trends by sex and age and Histological types of 37 cases of human thyroid carcinomas from the HMIMV of Rabat, Morocco

	Female	Male	PTC	FTC	ATC
**Age (year)**	42.6 ± 2.1	50.9 ± 6.1			
**Range of age (year)**	(23–75)	(25–64)			
**Number of cases**	26/37	8/37	(29/37)	(7/37)	(1/37)
%	70%	22%	78%	19%	3%

**Note**: 3 thyroid tumor blocks are issued from patients that the gender was not mentioned in the archived clinical registries.

Based on histological type classification, we evaluate the frequency of PTC, FTC and ATC in this cohort from the Anatomic Pathology and Histology Service of the HMIMV in Rabat which is at our knowledge the first experience from Morocco. We find that PTC is more frequent accounting for 78% followed by FTC (19%) and finally ATC (3%); and this profile distribution is in concordance with the international trend 1.

### BRAF^V600E^ mutation

**Fig. 1:**
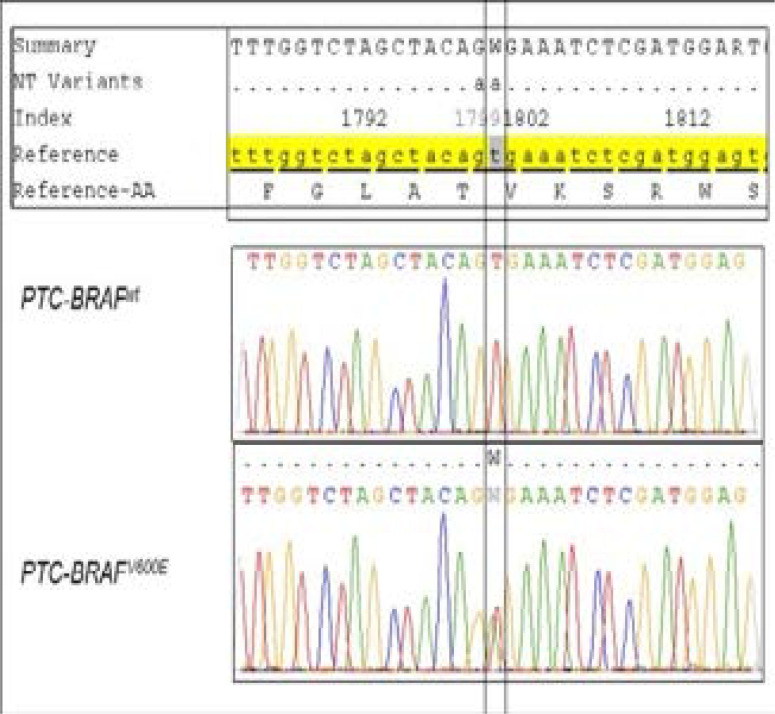
example of sequencing electropherogram with BRAF codon wild-type and V600E heterozygous in tumors from two PTC. PTC-BRAF^wt^: PTC FFPE tumor showing no mutation V600E in BRAF. PTC-BRAF^V600E^: PTC FFPE tumor showing the presence of the mutation V600E in BRAF.

We have used DNA from BCPAP cell line, which are BRAF^V600E^ homozygous, to validate BRAF^V600E^ detection method (data not shown). The presence of BRAF^V600E^ in the FFPE blocs is detected using the same method. Figure 1 shows electropherogram of BRAF wild-type PTC and heterozygous BRAF-mutated PTC (Fig. 1).

In our study, BRAF^V600E^ hot spot mutation is detected exclusively in PTC and any mutation has been found in FTC (Table 2). This first report from this single-institution study in Morocco is in concordance with international literature concerning the specificity of BRAF^V600E^ for PTC compared with FTC 1; 8. This mutation can occur rarely in follicular variant of PTC ‘FVPTC’ 19, but in our retrospective study all PTC have only a classical forms. According to the literature and unlike the most common BRAF mutation observed in PTC, K601E mutation in the Exon 15 of BRAF has been described in some cases of both classical FTC 36 and FVPTC 19; and in our study any BRAF^K601E^ was detected in FTC (Table 2).

**Table 2:** *BRAF^V600E^* detection in *37 cases of human thyroid carcinomas from the HMIMV of Rabat, Morocco*

	PTC	FTC	ATC
**Number of cases**	29	7	1
**Number of *BRAF^V600E^* positive**	8	0	0
*% in each histological type*	28%	0%	0%
**Number of *BRAF^K601E^* positive**	0	0	0
**Number of cases with Lymph node** **metastases**	5	0	0
*% in each histological* *type*	17.24%	0%	0%

We count BRAF^V600E^ in 8 PTC out 29 PTC accounting for 28% (Table 2). BRAF^V600E^ mutation was detected at different frequencies in PTCs (30 to 90% in PTCs): 15 from 42 PTC in the North India (35.7%; direct sequencig); in 170 from 543 PTCs in China (31.3%; direct sequencing); 84 from 266 PTC in Serbia (31.6% ; direct sequencing); 12 from 25 PTC in Poland (48% ; AS-PCR/SSCP and direct sequencing); 242 from 631 PTC in Japan (38.4%; direct sequencing); 190 from 211 PTCs in Korea (90%; PNA clamp real-time PCR)^8^; ^9^; ^10^; ^11^; ^12^; ^15^.

The prevalence of BRAF^V600E^ mutation in our study (28%, Table 2) falls within the range of international trends (30% to 90%) reported in distinct worldwide geographic regions. These results could indicate no apparent geographic specificity regarding the molecular statut of BRAF^V600E^ mutation in our cohort. Other studies must be carried out including different regions from Morocco, focused on a large cohort and using pyrosequencing even if a good concordance (94%) between the both sequencing methods (direct sequencing vs pyrosequencing) has been reported 25.

**Fig. 2:**
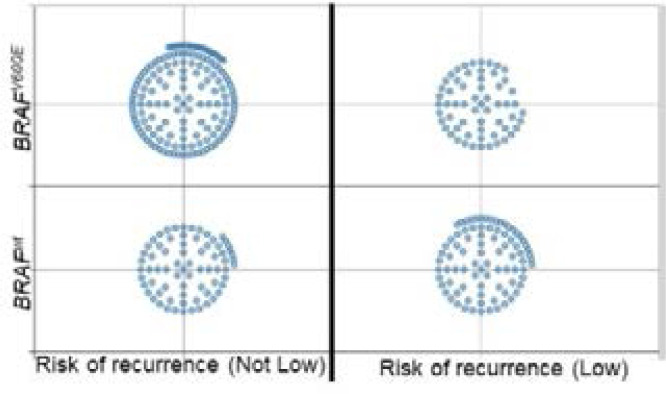
The association of *BRAF^V600E^* mutation and risk of recurrence in PTCs from TCGA^*5*^. BRAF^V600E^ mutation is detected in PTC with low and high risk of recurrence and its prevalence is more important in PTC with poor prognosis.

In this retrospective study, 5 PTC patients from 29 showed lymph node metastasis (Table 2) in which 3 tumors are harboring BRAF^V600E^ mutation. This finding is in concordance with the already reported association between BRAF^V600E^ oncogene and the worse clinical prognostic of PTC as well as the extrathyroidal invasion^4^; ^5^; ^7^. We explore a large cohort of 496 PTCs from The Cancer Atlas Genome (TCGA) 5 and we observe that BRAF^V600E^ mutation is more prevalent in thyroid tumors when the risk of recurrence is not low (Fig. 2). This observation confirms the reported positive correlation between the presence of this hot spot mutation and the poor prognosis and aggressively of thyroid tumors (BRAF^V600E^ positive). However, we observe also the wild type form of BRAF when the risk of recurrence is not low (Fig.2) highlighting the need of the identification of supplementary marker(s), downstream BRAF^V600E^, that could refine the clinical use of BRAF^V600E^ in the management of thyroid carcinomas. In fact, the PTC tumors harbouring BRAF^V600E^ (PTC-BRAF^V600E^ ) are heterogeneous and this mutation is detected in two subgroups of PTC showing different TDS (Thyroid Differentiation Score) and distinct level of ERK signature 5. Interestingly, the more aggressive PTCs-BRAF^V600E^ are highly dedifferentiated (TDS is negative) and showed a high level of activation of ERK^5^. Recently, Azouzi et al., demonstrated that BRAF^V600E^ down-regulated NIS in two human thyroid tumor cell lines through a redox mechanism involving the NADPH oxidases NOX4^22^. The exploration of data from TCGA showed that NOX4 is positively correlated with 1) dedifferentiation of PTCs-BRAF^V600E^, and with 2) ERK activation 22

Taken together, more deep investigations are needed to clarify how thyroid dedifferentiation, ERK activation level and NOX4 expression level could be used in the clinical routine as a panel of markers for the individual management of thyroid carcinoma harbouring BRAF^V600E^ mutation.

Although the importance of this first study in Morocco concerning the non-negligible prevalence of BRAF^V600E^ oncogene in thyroid tumors, studies including more hospital centers from different geographic region are needed to precise the national prevalence of BRAF^V600E^ in thyroid tumors as well as its possible clinical significance.
